# Assessment of delivered dose in prostate cancer patients treated with ultra-hypofractionated radiotherapy on 1.5-Tesla MR-Linac

**DOI:** 10.3389/fonc.2023.1039901

**Published:** 2023-01-19

**Authors:** Lin-Rui Gao, Yuan Tian, Ming-Shuai Wang, Wen-Long Xia, Shi-Rui Qin, Yong-Wen Song, Shu-Lian Wang, Yu Tang, Hui Fang, Yuan Tang, Shu-Nan Qi, Ling-Ling Yan, Yue-Ping Liu, Hao Jing, Bo Chen, Nian-Zeng Xing, Ye-Xiong Li, Ning-Ning Lu

**Affiliations:** ^1^ Department of Radiation Oncology, National Cancer Center/National Clinical Research Center for Cancer/Cancer Hospital, Chinese Academy of Medical Sciences and Peking Union Medical College, Beijing, China; ^2^ Department of Urology, National Cancer Center/National Clinical Research Center for Cancer/Cancer Hospital, Chinese Academy of Medical Sciences and Peking Union Medical College, Beijing, China; ^3^ GCP Center/Clinical Research Center, National Cancer Center/National Clinical Research Center for Cancer/Cancer Hospital, Chinese Academy of Medical Sciences and Peking Union Medical College, Beijing, China; ^4^ Department of Urology and State Key Laboratory of Molecular Oncology, National Cancer Center/National Clinical Research Center for Cancer/Cancer Hospital, Chinese Academy of Medical Sciences and Peking Union Medical College, Beijing, China

**Keywords:** prostate cancer, ultra-hypofractionated radiotherapy, MR-guided adaptive radiotherapy, beam-on, dosimetry analysis

## Abstract

**Objective:**

To quantitatively characterize the dosimetric effects of long on-couch time in prostate cancer patients treated with adaptive ultra-hypofractionated radiotherapy (UHF-RT) on 1.5-Tesla magnetic resonance (MR)-linac.

**Materials and methods:**

Seventeen patients consecutively treated with UHF-RT on a 1.5-T MR-linac were recruited. A 36.25 Gy dose in five fractions was delivered every other day with a boost of 40 Gy to the whole prostate. We collected data for the following stages: pre-MR, position verification-MR (PV-MR) in the Adapt-To-Shape (ATS) workflow, and 3D-MR during the beam-on phase (Bn-MR) and at the end of RT (post-MR). The target and organ-at-risk contours in the PV-MR, Bn-MR, and post-MR stages were projected from the pre-MR data by deformable image registration and manually adapted by the physician, followed by dose recalculation for the ATS plan.

**Results:**

Overall, 290 MR scans were collected (85 pre-MR, 85 PV-MR, 49 Bn-MR and 71 post-MR scans). With a median on-couch time of 49 minutes, the mean planning target volume (PTV)-V_95%_ of all scans was 97.83 ± 0.13%. The corresponding mean clinical target volume (CTV)-V_100%_ was 99.93 ± 0.30%, 99.32 ± 1.20%, 98.59 ± 1.84%, and 98.69 ± 1.85%. With excellent prostate-V_100%_ dose coverage, the main reason for lower CTV-V_100%_ was slight underdosing of seminal vesicles (SVs). The median V_29 Gy_ change in the rectal wall was -1% (-20%–17%). The V_29 Gy_ of the rectal wall increased by >15% was observed in one scan. A slight increase in the high dose of bladder wall was noted due to gradual bladder growth during the workflow.

**Conclusions:**

This 3D-MR–based dosimetry analysis demonstrated clinically acceptable estimated dose coverage of target volumes during the beam-on period with adaptive ATS workflow on 1.5-T MR-linac, albeit with a relatively long on-couch time. The 3-mm CTV-PTV margin was adequate for prostate irradiation but occasionally insufficient for SVs. More attention should be paid to restricting high-dose RT to the rectal wall when optimizing the ATS plan.

## Introduction

External beam radiotherapy (EBRT) is one of the recommended treatment modality for localized prostate cancer (PCa). With the evolution of RT technique and radiobiological progress, the EBRT course had decreased from nearly 2 months with conventional fractionation to within 1–2 weeks with ultra-hypofractionated RT (UHF-RT). Although the PACE-B trial (administration of 36.25 Gy in five fractions over 1–2 weeks) did not demonstrate any difference in acute toxicities (1), another randomized controlled trial, HYPO-RT-PC (administration of 42.7 Gy in seven fractions over 2.5 weeks) identified more severe urinary side-effects at 1 year in the UHF-RT group (2).

Inter- and intra-fractional variability of target volumes and organs at risk (OARs) deformation and shifting called into question the safety of further dose escalation and UHF-RT for PCa. Contrary to the commonly used volumetric modulated arc therapy (VMAT), and intra-fractional motion monitoring or repeated static imaging in the PACE-B trial, the majority (80%) of patients in the HYPO-RT-PC trial were treated by 3-dimensional conformal radiotherapy and position control was not feasible during fraction delivery (2). Even with cone-beam CT (CBCT) registration, the prostate target coverage was only 61.9-62%, which means online adaptive RT is needed for approximately one-third of the treatment fractions (3, 4). In addition, the resolution of CBCT images was generally low for prostate registration (4). Moreover, a fiducial marker or electromagnetic transponder insertion can improve the registration accuracy (4), but is inconvenient to patients due to invasiveness, potential pain, bleeding, and marker shifting. Furthermore, neither of the above-mentioned registration steps could compensate for the prostate (5–7) and seminal vesicle (SV) (8) deformations, nor the OARs (mainly bladder and rectum) motion.

Magnetic resonance (MR)-guided radiotherapy (MRgRT) is a milestone in the progress of RT technique. It not only affords improved soft-tissue resolution for registration but also brings online adaptive RT into clinical practice. With the integration of 1.5-Tesla MR into 7-MV linac, the Elekta 1.5-T MR-linac provided online Adapt-To-Position (ATP) and Adapt-to-Shape (ATS) workflows. The ATS workflow can meet all the above requirements of PCa UHF-RT by online target editing and optimizing plan from fluence optimization (9). Furthermore, real-time 2D cine MR can be used to monitor the motion, and 3D high-resolution magnetic resonance imaging (MRI) can be acquired during the beam-on period. Both these approaches allow for motion control and help to achieve high-precision RT delivery (10). However, the current online adaptive procedure is time-consuming, which makes many researchers concerned about the accuracy of the delivered dose, especially the dosimetric effects on the target and OARs due to intra-fractional motion.

De Muinck Keizer et al. firstly reported prostate intra-fraction motions during each ATS session and dose reconstruction using cine MR dynamics, which was determined with a previously validated soft-tissue contrast–based tracking algorithm (11, 12). For each fraction, the treatment delivery record was generated by proportionally splitting the plan into 11s intervals based on the delivered monitor units (13), which could possibly affect the actual delivered dose. Hence, the purpose of this study was to estimate the delivered dose for targets and OARs by dosimetry analysis based on high resolution 3D-MR aquisitions, including pre-, position verification (PV-), beam-on (Bn-), and post-3D-MR scans, of each adaptive RT session for PCa patients treated on 1.5-T MR-linac.

## Materials and methods

### Patient eligibility

A prospective observational study with regular follow-up was initiated for PCa in 2019 to investigate the feasibility, tolerability, and toxicity profiles of UHF-RT on 1.5-T MR-linac (NCT05183074, ChiCTR2000033382). The risk group was defined per the National Comprehensive Cancer Network (NCCN) v.1.2019 edition. For this study, dosimetry data were collected from 17 consecutive patients with localized low-, intermediate- to selective high-risk PCa (**Table S1**).

### Target volume delineation and reference plan

Simulation CT (slice thickness = 3 mm) and MR scans (Contrast enhanced T1-weighted imaging, Fast spin echo T2-weighted imaging and diffusion weighted imaging, slice thickness = 3 mm) were acquired and registered for contouring and reference planning. About 1 hour before simulation and each RT session, the patients were instructed to empty the rectum and bladder and asked to drink 300 to 500 ml of water in 15 to 20 minutes to ensure slow filling of the bladder, in consideration of the long on-couch time of the ATS workflow. Target delineation was defined as per EORTC-ACROP contouring guidelines (14). The clinical target volume (CTV) was defined as the whole prostate for low-risk disease (N = 1) and the whole prostate with a 3-mm margin (0 mm posteriorly) for patients (N = 16) with a potential extraprostatic extension (EPE) rate of 20% or higher per the Partin tables. The proximal 1 cm SVs were included for patients with an SV involvement rate of 15% or higher (N = 11), and the whole SV was included for patients with minimal T3b (N = 1). The planning target volume (PTV) was derived from the CTV plus a uniform 3-mm margin. For intermediate- to high-risk disease (N = 16), a simultaneous boost of CTV 40 was defined as prostate with contraction of 1 mm. Rectal wall and bladder wall were defined as the 3 mm-inner rings of the rectum and bladder, respectively.

The prescription doses of PTV and CTV 40 were 36.25 Gy and 40 Gy, respectively, in five fractions delivered every other day, with a total course of 10 to 12 days. The target volume dose prescription and OARs constraints for UHF-RT are listed in **Table S2**. Then a reference plan was generated using the Monaco (v5.40, Elekta AB, Stockholm, Sweden) planning system, with 7 to 10 beams and less than 80 segments (<120 segments was acceptable for complicated plans).

### Online ATS workflow and image acquisition

**Figure 1:**
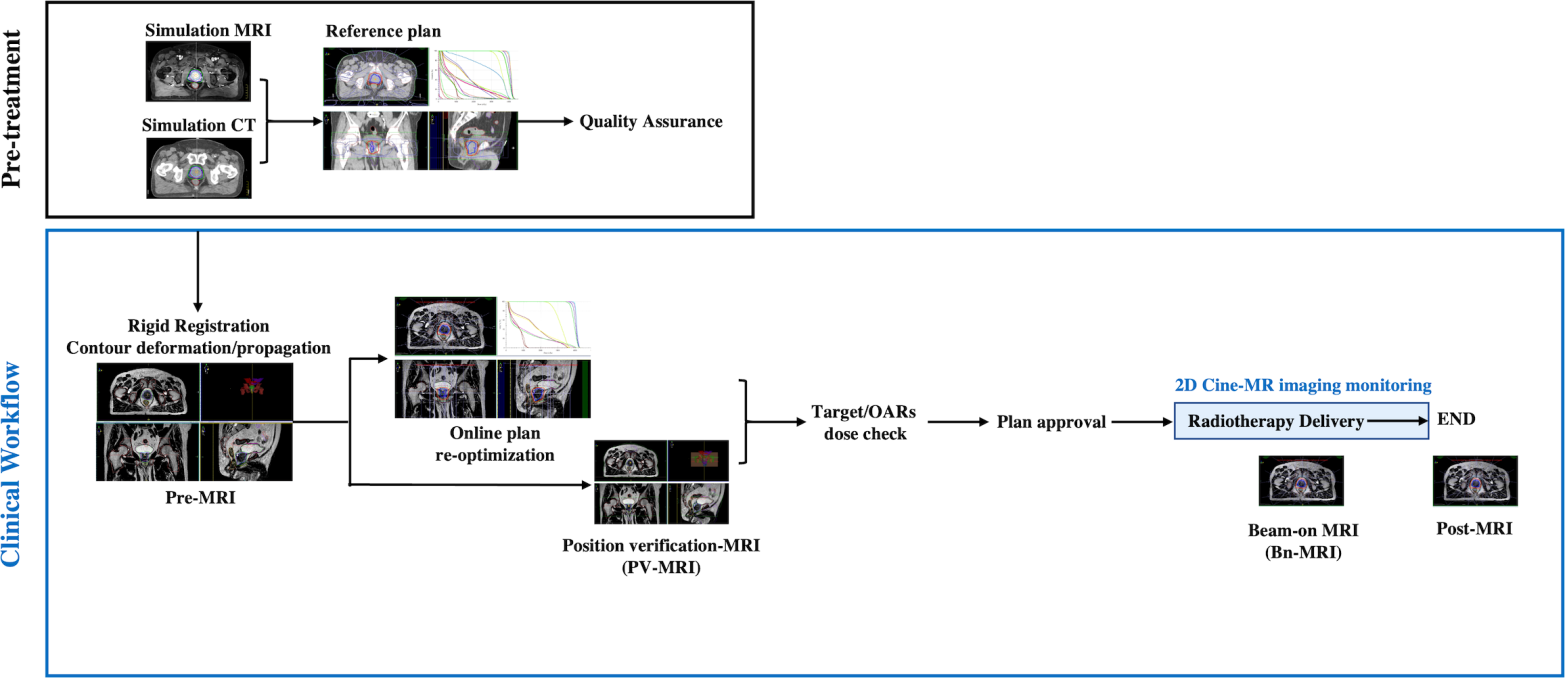
Pre-treatment and clinical workflow. Simulation MRI, MRI acquired for reference plan; usually scanned 1 to 2 weeks before treatment; Pre-MRI, MRI acquired each treatment day for adaptive plan optimization; OARs, Organs at risk; PV-MR, Position verification-MR.

The image acquisition procedure is listed in **Figure 1**. During each fraction, an initial (pre-MR) scan was acquired after set-up using a T2-weighted 3D sequence with a duration of 6 minutes for the first 12 patients and that of 2 minutes thereafter. After rigidly registering the pre-MR data to simulation CT or previous pre-MR image, contours were automatically deformed to the pre-MR image and manually adapted by the physician, followed by full plan re-optimization in the Monaco system starting from fluence optimization (9). The pseudo-CT is generated using the bulk electron density assignment strategy, that is, the inside of each region of interest (ROI) on the MR image is filled with the mean relative electron density of the corresponding ROI on the reference CT image according to the user-specified layer order. Before the end of plan reoptimization, a PV-MR scan was acquired. If the CTV was still within the PTV on the PV scan and the rectum did not move ventrally, the ATS plan was accepted and treatment delivery with real-time cine MR was started.

For the first seven patients, 2D cine MR images were continuously collected during the “beam-on” period, owing to concerns about unexpected target and OAR moving. The delivery will be interrupted if the prostate moved out of the PTV or the rectum moved ventrally. From the eighth patient, if the position of all the organs was stable, 2D cine MR monitoring was stopped and a Bn-MR scan was acquired using a T2-weighted 3D sequence with a duration of 2 minutes. Directly after RT delivery, another post-MR T2-weighted 3D sequence scan was acquired. The procedure was well tolerated for the majority of sessions; however, no post-MR scan was acquired in three sessions for two patients because of their bladders being excessively full. An extended workflow was used in three sessions because of rectum motion, in another three sessions because of an overfilled bladder, and in one session because of SVs moving out of CTV (one with another ATP and six with another ATS workflow).

### Image fusion and re-planning on each MR scan for dose calculation

By image registration and propagation of anatomical contours, the targets and OARs of the ATS plan for each session were transferred to the corresponding PV-, Bn-, and post-MR scans, respectively. The same radiation oncologist edited the targets and OARs manually to ensure contouring consistency if necessary. A senior radiation oncologist reviewed all the contours. The dose distribution for the online ATS plans was recalculated on each pseudo-CT scan derived from each MR scan by using the “original segments” mode. The dose metrics were evaluated for the adapted ROIs. For each fraction, the volumes of the clinical targets and OARs, as well as the re-computed doses on different MR scans were compared with the corresponding parameters of the online ATS plan, instead of comparing the cumulative dose of five fractions with that of the original ATS plan.

### Statistical analysis

SPSS 25.0 (IBM Corp., Armonk, NY, USA) was used for statistical analysis. Continuous variables are presented as the mean ± SD, median (range), 95% confidence interval (CI), or frequencies with percentages depending on their distribution. Generalized estimating equation was used to compare the variables on different scans for each fraction. Differences were defined as significant when the p-value was <0.05.

## Results

### Patients’ characteristics

Patients’ (N=17) characteristics are shown in **Table S1**. The median patient age was 75 (58–87) years. The baseline prostate-specific antigen (PSA) level was ≤10 ng/ml in five (29.4%) patients, 10–20 ng/ml in five (29.4%) patients, and ≥20 ng/ml in seven (41.2%) patients. Per the NCCN risk grouping, there were 2 (11.8%), 12 (70.6%), and 3 (17.6%) patients with low-, intermediate-, and high-risk diseases, respectively. The median prostate volume was 42.48 (28.86–64.14) cc.

### MRI for analysis

**Figure 2:**
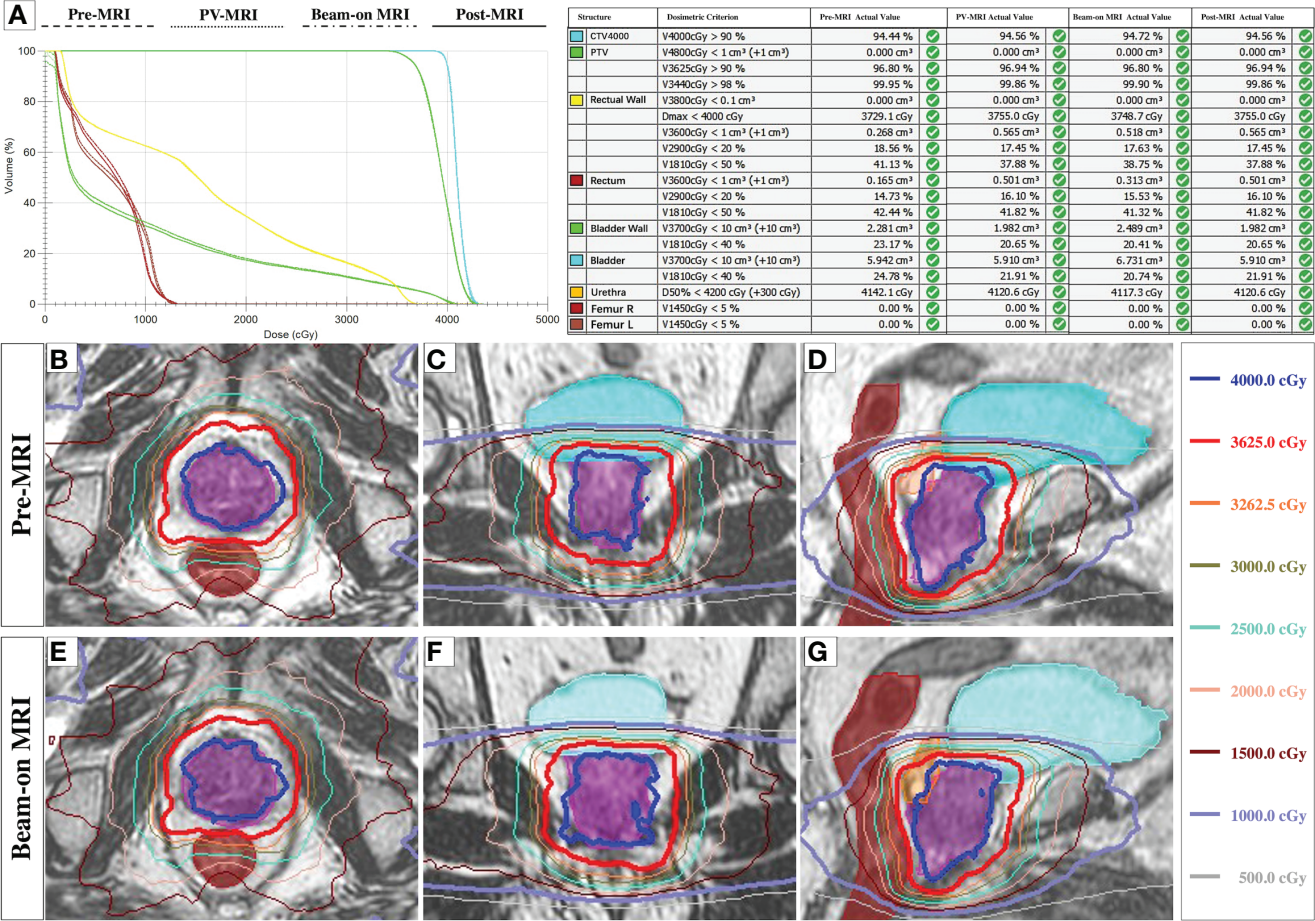
The dose distributions on each MRI scans. **(A)**: The representative DVH plot with four plans and the dose metrics on each MRI scans after re-planning in one fraction. **(B–G)**: The representative dose distributions of three planes of ATS plan on Pre-MR **(B–D)** and Beam-on MR scan **(E–G)**.

In total, 290 1.5-Tesla high-resolution MRIs from 85 fractions of 17 consecutive patients were used for dosimetry analysis, including 85 pre-, 85 PV-, 49 Bn-, and 71 post-MR scans, respectively. Beam-on 3D-MR scans were collected from 49 fractions of 10 patients because for one session, we observed rectum gas bubbles and used continuous 2D cine MR for monitoring. Post-MR scans were not acquired for three sessions of two patients because of their bladders being too full, and the remaining 11 post-scans of five patients failed to transmit to the Monaco system. An example of the dose distributions on each MR scans after re-planning was shown in **Figure 2**.

### Target dose coverage

The median on-couch time was 49 (24–78) minutes. Comparison of the target and OARs volumes and volume differences relative to those in the corresponding ATS plan (based on pre-MR scans) are shown in **Table S3**. For each fraction, the target volume differences of the prostate and CTV on different scans were less than 3.0 cc, indicating good consistency of target contouring.

**Figure 3:**
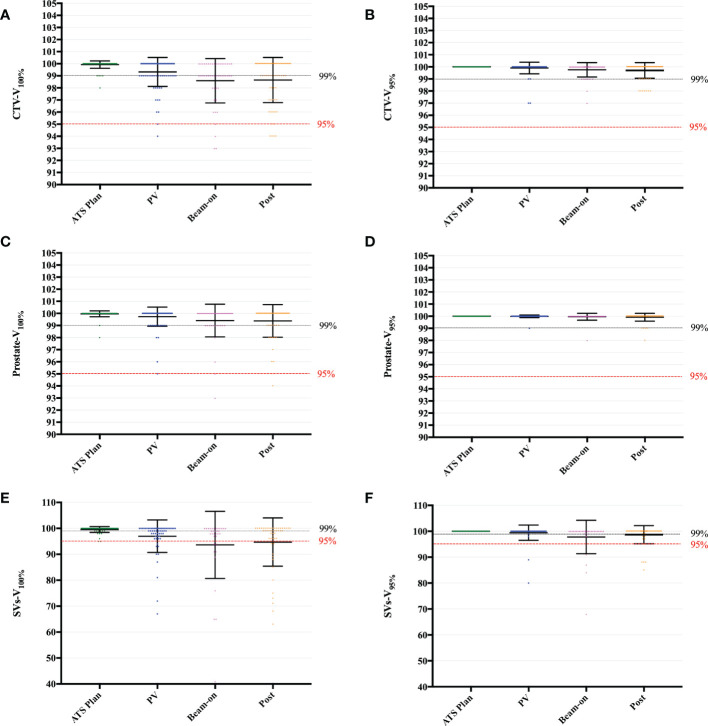
Boxplot of V_100%_ and V_95%_ values to the CTV **(A, B)**, Prostate **(C, D)** and SVs **(E, F)**, calculated by the daily ATS plan dose on the PV-, Bn- and post-MR scan for each session and patient. Individual data points are shown as dots. The mean ± SD are shown as the error bars.

The planning targets of all fractions, calculated by the daily ATS plan dose in PV-, Bn- and post-MR scans were shown in the **Figure S1**. The mean PTV-V_95%_ (V_34.4Gy_) of all scans was 97.83 ± 0.13% (**Figure S1B**). On 27/290 (9.3%) scans, the PTV-V_95%_ was less than 95% (**Figure S1B**). Furthermore, the mean CTV-V_100%_ (V_36.25Gy_) of all scans was 99.21 ± 0.09%, and that of the ATS plan and PV-MR, Bn-MR, and post-MR phases, respectively, was 99.93 ± 0.30%, 99.32 ± 1.20%, 98.59 ± 1.84%, and 98.69 ± 1.85% (all p < 0.001; **Figure 3A**). Interestingly, the average CTV-V_100%_ (V_36.25Gy_) of each phase was all covered by 98% of the prescribe dose during treatment. With excellent dose coverage of prostate-V_100%_ (V_36.25Gy_) (**Figure 3C**), the main reason for lower CTV-V_100%_ (V_36.25Gy_) was slight underdosing of SVs (**Figure 3E**).

The V_95%_ of CTV (**Figure 3B**), the prostate (**Figure 3D**), and SVs (**Figure 3F**) were shown in **Figure 3**. Among the 31 scans on which the SV-V_36.25Gy_ was less than 95%, the SVs-V_100%_ (SV-V_36.25Gy_) was between “=90%” and 95% on 6.3% (13/206) scans, between “=85%” and 90% on 2.4% (5/206) scans, between “=75%” and 85% on 1.9% scans (4/206), between “=60%” and 75% on 3.9% scans (8/206) and only 41% on one scan, respectively (**Figure S1A**). The corresponding SVs-V_95%_ (SV-V_34.4Gy_) was between “=90%” and 95% on 0.5% (1/206) scans, between “=85%” and 90% on 3.0% (6/206) scans, between “=75%” and 85% on 1.0% (2/206) scans, and less than 75% on one scan (**Figure S1B**). Furthermore, SV-V_34.4Gy_ of less than 95% was found in 6 fractions of 3 patients.

**Figure 4:**
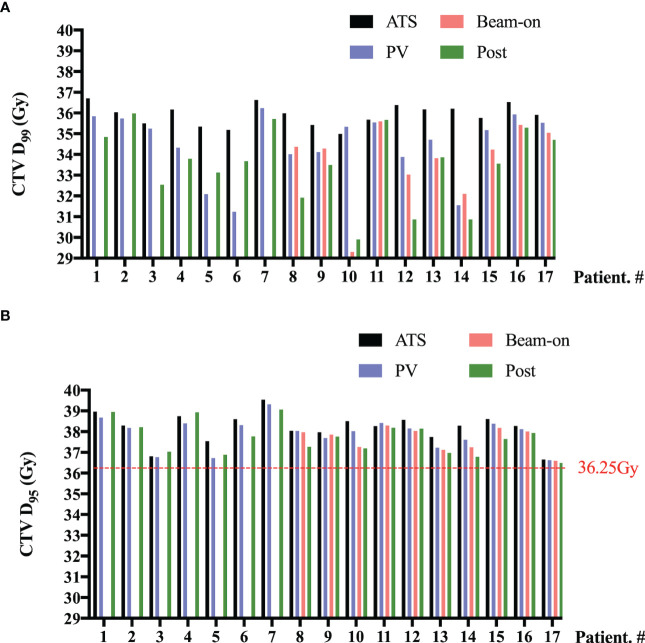
Per-patient D_99%_ **(A)** and D_95%_ **(B)** values to the clinical target volume (CTV) summed by five fractions of pre- (ATS plan), PV-, Bn- and post-MR scans. No beam-on scans were acquired for the first 7 patients due to concerns about unexpected target and OAR moving.

As shown in **Figure 4**, we also summed the CTV-D_99%_ (**Figure 4A**) and CTV-D_95%_ (**Figure 4B**) values of five fractions on a per-patient basis for pre-, PV-, Bn-, and post-MR scans, respectively. Although there were 12 patients with SV underdose, the sum of CTV-D_95%_ on each MR scan was higher than the prescription dose (36.25 Gy) for all 17 patients (**Figure 4B**).

### OARs

**Figure 5:**
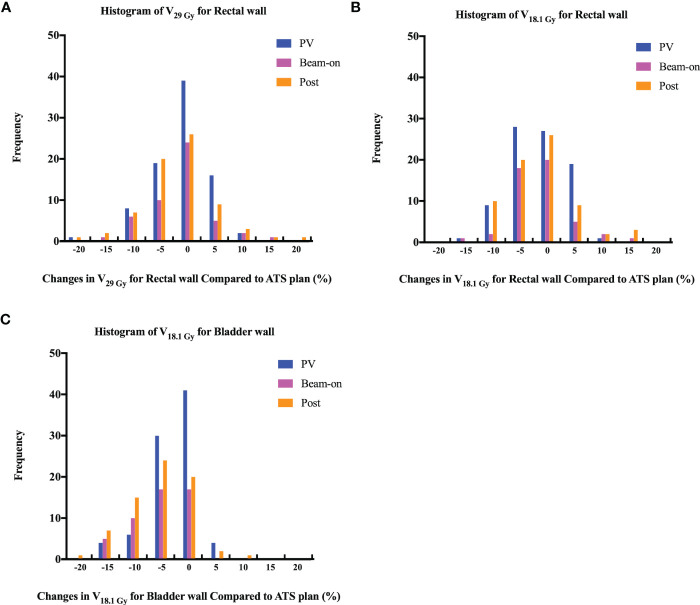
Histogram of changes in volume receiving 29 Gy (**A**, V_29 Gy_) or 18.1 Gy (**B**, V_18.1 Gy_) dose for rectal wall and 18.1 Gy **(C)** dose (V_18.1 Gy_) for bladder wall among PV-, Bn- and post-MR scans compared to ATS plans.

The volumes of the rectum fluctuated during treatment, with mean variation of 1.59 cc, 2.37 cc, and 1.37 cc on the PV-MR, Bn-MR and post-MR scans, respectively (**Table S3**). In comparison to the values of ATS plan, the estimated delivered dose to the rectal wall during the whole workflow also varied (**Table 1**), with a mean variation V_38Gy_ of 0.23 ± 0.28 cc on PV-MR, 0.41 ± 0.51 cc on Bn-MR, and 0.39 ± 0.52 cc on post-MR, and a mean variation V_36Gy_ of 0.27 ± 0.57 cc on PV-MR, 0.39 ± 0.71 cc on Bn-MR, and 0.30 ± 0.68 cc on post-MR. There is no statistical difference between mean V_29 Gy_ or V_18.1 Gy_ in the rectal wall of ATS plan and that of PV-MR, Bn-MR, and post-MR phases, respectively (p = 0.882, 1.000 and 0.587 for V_29 Gy_; p = 0.221, 1.000 and 0.363 for V_18.1 Gy_). The changes in the V_29Gy_ and V_18.1Gy_ of the rectal wall in comparison with the ATS plans are shown in **Figures 5A, B**. The median V_29 Gy_ change in the rectal wall was -1% (-20%–17%). An increase of >15% in V_29Gy_ was only observed in one scan (1/205, 0.5%). No fraction showed an increase of >15% in the V_18.1Gy_ of the rectal wall. The V_29Gy_ of the rectal wall (**Figure 5A**) showed an increase of 5%–15% in 21.2% (18/85), 16.3% (8/49), and 18.3% (13/71) of the PV-, Bn-, and post-MR scans, respectively, and the corresponding values for an increase of 5%–15% in V18.1Gy (**Figure 5B**) were 23.5% (20/85), 16.3% (8/49), and 19.7% (14/71), respectively.

**Table 1:** Dose metrics for the original ATS plan and re-computed plans on PV-MR, beam-on MR and post-MR scans.

Dose metrics		ATS Plan (Mean±SD)	PV (Mean±SD)	Beam-on (Mean±SD)	Post (Mean±SD)
Target coverage					
CTV4000					
	V_100% (40 Gy)_	95.90±0.26	93.46±0.44	92.14±0.58	91.70±0.61
	V_42.5Gy_ (cc)	4.26±5.38	4.55±5.68	3.58±4.45	4.34±5.61
PTV					
	V_100% (36.25Gy)_	95.27±1.67	92.60±3.14	91.53±3.23	91.47±3.80
	V_95% (34.4 Gy)_	99.27±0.75	97.69±2.03	97.04±2.29	96.87±2.57
OAR metrics					
Rectal wall					
	D_max_	38.35±1.09	38.71±2.22	38.77±2.27	38.71±2.59
	V_38 Gy (cc)_	0.05±0.08	0.28±0.49	0.36±0.56	0.40±0.80
	V_36 Gy (cc)_	0.59±0.39	0.86±1.00	0.94±1.03	0.92±1.15
	V_29 Gy_	16.45±4.99	15.25±6.96	16.33±6.44	15.47±8.26
	V_18.1 Gy_	34.92±6.72	33.32±8.61	35.12±7.29	34.20±8.91
Bladder wall					
	V_37Gy_ (cc)	2.44±1.15	2.86±1.50	3.09±1.34	3.27±1.85
	V_18.1 Gy_	23.80±8.98	20.53±9.41	19.49±8.05	18.84±7.43
Intestine	D_max_	2.32±2.30	1.81±1.86	1.75±1.92	1.93±2.23
Colon	D_max_	5.10±4.37	3.87±3.19	4.11±3.32	3.78±3.00
Femur L	V_14.5 Gy_	0.94±2.57	0.99±2.67	1.41±3.28	1.10±2.85
Femur R	V_14.5 Gy_	0.73±1.48	0.73±1.30	0.63±1.22	0.71±1.60

In contrast, the bladder volume gradually increased with time, with mean variation of 83.96 cc, 136.75 cc, and 140.36 cc, respectively (**Table S3**). As the volume of the bladder increased, the bladder volume receiving high dose increased slightly. The mean V_37Gy_ of the ATS plan and PV-MR, Bn-MR, and post-MR phases was 2.44 ± 1.15 cc, 2.86 ± 1.50 cc, 3.09 ± 1.34 cc, 3.27 ± 1.85 cc, respectively (**Table 1**). The mean variation V_18.1Gy_ was -0.03 ± 0.12 cc on PV-MR, -0.06 ± 0.03 cc on Bn-MR, and -0.05 ± 0.03 cc on post-MR, respectively, due to gradual growth of the bladder over the workflow. A V_18.1Gy_ increase of 10% was only observed in one scan (1/205, 0.5%), while an increase of >5% was only observed in 4.7% (4/85), 0%, and 4.2% (3/71) of the PV-, Bn- and post-MR scans, respectively (**Figure 5C**).

The dose metrics are summarized in **Table 1**. The D_max_ of the colon and intestine and the V_14.5Gy_ of femur L/R were also evaluated.

## Discussion

To our knowledge, this is the first study assessing the delivered dose to targets and OARs of online adaptive UHF-RT for PCa patients based on high resolution 3D beam-on and post-treatment MRIs on a 1.5 T MR-linac. Our study demonstrated clinically acceptable estimated dose coverage of target volumes during the beam-on period with an adaptive ATS workflow, with a slight increase of rectal wall volume receiving high dose and a gradual reduction of the bladder dose. The 3-mm CTV-PTV margin applied in our study has been shown to be sufficient for the prostate and may be insufficient for a small portion of SVs.

The potential effects of inaccurate delivery of radiotherapy doses due to inter- and intra-fractions on treatment efficacy and/or toxicity of normal tissues has long been a concern associated with radical radiotherapy. Several strategies have been adopted to decrease these potential effects, with CBCT w/o fiducial markers being the most widely used approach. Peng et al. observed target underdosing in approximately one-third of the treatment fractions with CBCT using prostate alignment, with the Prostate-V_100%_ decreasing by >15% in 4.3% of the fractions and by 3%–15% in 18.0% of the fractions (3). CBCT with insertion of fiducial markers or Calypso with electromagnetic transponder tracking will improve the treatment accuracy to levels comparable to those of MRgRT (4, 7, 15), but the invasiveness of the insertion procedures has made them difficult to be widely used in clinical practice. Moreover, none of the non-adaptive radiotherapies can offset the prostate volume changes during the treatment course (16), which would be more significant with extreme hypo-fractionation schedules, and could be associated with the prostate continuous swelling during the whole course observed by Gunlaugsson et al. (17). With the online ATS workflow, we re-contoured and re-optimized the plan for each session. The mean dose coverage of Prostate-V_100%_ was 99.66 ± 0.06%, and the Prostate-V_100%_ did not decrease by >10% on any scan in our study, demonstrating greater accuracy of dose delivery of adaptive RT.

The adaptive workflow provided by MRgRT offers the potential to characterize and track anatomy variations, and ultimately realize real-time plan adaptation. This could offer the opportunity for reducing CTV-PTV margins, and particularly suitable to prostate reirradiation with the need to deliver high doses in a small volume with maximum sparing of pelvic OARs (18, 19). Although both ATP and ATS workflow available for adaptation, the study investigating dosimetry analysis of 100 fractions of 20 PCa patients by our team showed that the ATP strategy could only meet the clinical requirements (relatively lower dose requirements with PTV-V90% achieving prescribed dose as goal) for 23 (23%) fractions, compared with 100 (100%) fractions by ATS strategy (20). Furthermore, some data also showed that only the optimization from fluence and segment could fit all requirements for prostate cancer (9).

The online adaptive workflow solved the problems with the inter-fraction motion, but accentuated the intra-fraction motion, especially with the obvious long on-couch time. Usually, 30–40 and 50–60 minutes were needed with the 2-minute and 6-minute MR scans (12, 21), respectively. Intra-fraction prostate motion assessed by Calypso electromagnetic beacons (22, 23), fluoroscopy (24) or 4D ultrasound (25) has previously been characterized as different categories, while more recent studies focusing on MRgRT which have monitored prostate motion over longer time periods have concluded differently (12).

Although the prostate motion was reported as different categories intra-fractionally with a larger range, it seems different as per studies aiming for adaptive RT in the MRgRT era. With a median of 49 (24–78) min of on-couch time in our study, we did observe a slight CTV dose reduction with time (**Figures 3A, B**). Similar findings had been reported by other studies based on dose reconstruction algorithms using beam-on 2D-cine MR (26) or cine MR dynamics (11), both of which adopted moderate fractionation schedules (60 Gy/20 fr or 62 Gy/20 fr). Menten et al. analyzed prostate intra-fraction motion and extrapolated the dose changes by processing MR-linac treatment log files and online 2D-cine MR, and concluded that the mean CTV-D_98%_ decreased by 1.1 Gy ± 1.6 Gy (26). The UMC Utrecht constructed a soft tissue tracking algorithm with cine MR dynamics, with a mean processing time of 10.7 ± 2.5 s per dynamic (11). By extracting the treatment log files and assigning them to the appropriate cine MR dynamic volumes, they deduced that the CTV-D_99%_ underwent a dose reduction of 2.2% ± 2.9% (11). Although a slight dose reduction was observed during the beam-on period in comparison with the ATS plan, the estimated dose delivered is still clinically acceptable. Research on prostate intra-fraction motion also demonstrated that the 95% CI of translation was within clinically applied margins of 5 mm by using cine MR dynamics (12), which was smaller than the data reported previously in the CBCT era (6–9 mm) (1, 2, 27). The main reasons for the small prostate motion and relatively stable dose coverage are as follows: first, the patients had been positioned on the couch for a relatively long time (27 minutes in de Muinck Keizer’s study (12) and 33 minutes in our study) before cine MR and treatment delivery in these adaptive MRgRT series, compared to usually less than 5 minutes in studies investigating motion with CBCT and VMAT. It was reported previously that the prostate intra-fraction motion reached saturation after approximately 30 min of on-couch time (12), which could probably explain the non-significant beam-on dose reduction with long on-couch time. Second, we advised patients to drink water more slowly during preparation to avoid quick bladder volume changes during on-couch, which could also account for the dose findings of acceptable target coverage.

Although a 3-mm margin from CTV to PTV seems to be adequate for the prostate, with prostate V_36.25Gy_ ≥ 95% for the 99% (204/206) scan (**Figure 3C**), it is not the case for SVs. The underdose (less than 95% of SVs-V_100%_) of SVs was observed on 31 scans collected from 18 fractions of 8 patients, which indicated that the intra-fractional SVs motion was a general problem. However, we also noticed that except for two patients, the SV-V_95%_ (SV-V_34.4Gy_) of the remaining 10 patients reached more than 95%, which indicated that the significant SV motion caused by long on-couch time is also patient-specific. Furthermore, one patient had some urine leakage after delivery due to too full bladder for two sessions, which caused worse underdose of SVs on Bn-scan compared with Post-scan (**Figures 3**, **4A**, Patient. #10 and **Figure S2**). The prostate and SVs have been shown to reveal independent motion characteristics, and SVs’ movement has been shown to correlate more with the movement of the bladder and rectum (28). The slow filling of the bladder and adequate preparation of the rectum in our study could mitigate the prostate motion due to bladder volume changes, but might not compensate for all SV motion. The maximal range (3.6-7.2 mm) of SV motion has been reported to occur in the superior-inferior dimension (8, 28, 29), and the range increases with treatment time (29). An intra-fractional SVs motion analysis of 15 PCa patients reported that the 5-mm margins provided 95% intra-fractional SV coverage in over 90% of fractions (8). De Muinck Keizer also reported that intra-fraction coverage probability of 99% can be achieved with 5 mm isometric expansion for the left and right SV on MR-linac (30).

The rectum volumes slightly fluctuated during the ATS workflow, although we asked all patients to empty the rectum with an enema before each session. The estimated delivered dose to the rectal wall was clinically acceptable (**Table 1**), which were similar or a little bit higher than the dose metrics of ATS plan (**Table S2**). Nevertheless, a rectal wall V_29Gy_ increase of >15% was only observed on one post-MR scan and a V_18.1Gy_ increase of >15% was not observed on any fractions (**Figures 5A, B**). In comparison with the data obtained using conventionally fractionated RT with CBCT, which reported 5.6% fractions of the rectum-V_45Gy_ increased by >15% (3), the online adaptive UHF-RT is safe for the rectum and delivers a more accurate dose. Moreover, during the treatment, gas pockets in the rectum were observed on 12.9% (11/85) of the scans, which were also reported in some studies (4, 10). The gas bubbles always occurred between the acquisition of the PV scan and the start of the cine MR acquisition and remained in place during dose delivery in most cases (11.8%, 10/85 scans). Only in one fraction, a gas bubble was observed on Bn-scan but disappeared on the post-scan. Nevertheless, the V_36.25Gy_ of CTV, prostate, and SVs for this patient were all 100% on each scan. Thus, continuous monitoring of target and rectum motion by cine MR is quite important for accurate dose delivery. Simultaneously, the continuous bladder volume increase caused a slight increase in bladder wall-mean variation V_37Gy_ (0.52 ± 0.58 cc) in a comparison with the ATS plan, and a reduction in bladder wall-mean variation V_18.1Gy_ (-0.05 ± 0.03 cc) conversely. The clinical findings also confirmed the estimated dose delivered to normal tissues. The rates of worst acute RTOG grade 2 or more severe genitourinary and gastrointestinal toxicities were 25% and 0% (unpublished data), as reported in our preliminary results.

This study had several limitations. The sample size was still small with only 17 patients. However, we collected dosimetry data on the PV-, Bn-, and post-MR scans, providing comprehensive data that can indicate the dose changes in all organs. Furthermore, it was still difficult to conclude the estimated delivered dose during the beam-on period by using the PV-, Bn-, and post-MR scans. In our study, we included 10 patients with beam-on 3D MR and recalculated the dose based on the beam-on 3D MR, which can provide a more accurate 3D representation of the prostate volume and position compared to that used by dose reconstruction approaches based on cine MR that collected MR images on certain slices. There are merits to using large field-of-view, high-resolution 3D MR acquisitions for dose estimation, however, the slow acquisition of 3D-MR images (approximately 3 min needed for the 2-min T2 MR), the low temporal resolution of the MR datasets, collecting data at certain time point instead of whole beam-on period and reliance on a bulk electron density assignment strategy would be a concern for the dose inaccuracy, and maybe tempo-wise less accurate than using continuous cine-MR dynamics (11, 12). Nevertheless, using similar high-resolution 3D-MR acquisitions with the adaptive ATS plan, our estimated dose should be reliable. In addition, we stopped the cine MR acquisition and collected beam-on 3D MR only when the target and rectum were stable on cine MR, therefore biasing the results towards good agreement with the planned doses. However, it seems that due to the low incidence of such cases, the impact of this limitation in practice is minimal. Furthermore, the dosimetry results demonstrated the reliability of our methods, which involved monitoring motion by cine MR and pausing delivery if necessary.

In conclusion, our study investigating the dose on beam-on 3D-MR scans for each session demonstrated that clinically acceptable estimated dose coverage of target volumes was achieved during the beam-on period with an adaptive ATS workflow on a 1.5-T MR-linac, despite the relatively long on-couch time. The 3-mm CTV-PTV margin applied in our study is sufficient for the prostate and may be inadequate for a very small portion of SVs. More attention should be paid to restricting the rectal wall high dose when optimizing the ATS plan.

## Data availability statement

The corresponding author has full access to all the data in the study and final responsibility for the decision to submit for publication. The data that support the findings of this study are available from H6WORLD platform (https://h6world.cn/signin) but restrictions apply to the availability of these data, which were used under license for the current study, and so are not publicly available. Data are however available from the authors upon reasonable request and with permission of H6WORLD platform. Requests to access the datasets should be directed to Ning-Ning Lu, Ning-Ning.Lu@hotmail.com.

## Ethics statement

The studies involving human participants were reviewed and approved by Independent Ethics Committee of Chinese Academy of Medicine Sciences (NCT05183074, ChiCTR2000033382). The patients/participants provided their written informed consent to participate in this study.

## Author contributions

N-NL, L-RG, YTian, M-SW, N-ZX, and Y-XL designed the study, analyzed the data and wrote the manuscript. N-NL, YTian, N-ZX, and Y-XL contributed to the study concept. N-NL, L-RG, YTian, M-SW, N-ZX, and Y-XL contributed to the study coordination. L-RG, N-NL, M-SW, and Y-XL performed the statistical analysis. All authors contributed to the article and approved the submitted version.

## Supplementary material

Supplementary Figure 1 Overviews of planning targets of all fractions, calculated by the daily ATS plan dose in PV-, Bn- and post-MR scans. 100% prescription dose **(A)** and 95% prescription dose **(B)**. Individual data points are shown as dots. The mean ± SD are shown as the error bars.

Supplementary Figure 2 Per-patient SVs-V_100%_ **(A)** and SVs-V_95%_ **(B)** mean values to the clinical target volume (CTV) summed by five fractions of pre- (ATS plan), PV-, Bn- and post-MR scans. Scar bars present the standard deviation (SD). No beam-on scans were acquired for the first 7 patients due to concerns about unexpected target and OAR moving. No SVs irradiation for Patient. #6, #7, #15 to #17.
