# Supplementary material for: Assessment of delivered dose in prostate cancer patients treated with ultra-hypofractionated radiotherapy on 1.5-Tesla MR-Linac
Source: Front Oncol. 2023 Jan 19;13:1039901. doi: 10.3389/fonc.2023.1039901 (PMC9893501; doi:10.3389/fonc.2023.1039901)
Supplement: Supplementary file 1 [file DataSheet_1.docx]

| **Table S1 Patient characteristics for whole group** | | |
| --- | --- | --- |
|  | **N=17** | **%** |
| **Age** |  |  |
| **Medium (range)** | 75 (range, 58–87) | |
| **PSA (ng/ml)** |  |  |
| **≤10** | 5 | 29.41 |
| **>10 & ≤20** | 5 | 29.41 |
| **>20** | 7 | 41.18 |
| **Risk group** |  |  |
| **Low** | 2 | 11.76 |
| **Intermediate** | 12 | 70.59 |
| **High** | 3 | 17.64 |

**Table S2 Target volume dose prescription and OAR constraints for the UHF-RT**

| **Target coverage** |  |  |
| --- | --- | --- |
| CTV4000 | V_100% (40 Gy)_ | >95% |
| CTV | V_100% (36.25 Gy)_ | >95% |
|  | V_95% (34.44 Gy)_ | >99% |
| PTV | V_100% (36.25 Gy)_ | >95% |
|  | V_95% (34.44 Gy)_ | >98% |
| **OAR constrains** |  |  |
| Rectal wall | V_38 Gy_ | <0.1cc |
|  | D_max_ | <40Gy |
|  | V_36 Gy_ | <1cc |
|  | V_29 Gy_ | <20% |
|  | V_18.1 Gy_ | <50% |
| Rectum | V_36 Gy_ | <1cc |
|  | V_29 Gy_ | <20% |
|  | V_18.1 Gy_ | <50% |
| Bladder wall | V_37 Gy_ | <10cc |
|  | V_18.1 Gy_ | <40% |
| Bladder | V_37 Gy_ | <10cc |
|  | V_18.1 Gy_ | <40% |
| Urethra | D_50%_ | <41Gy |
| Femur R/L | V_14.5 Gy_ | <5% |

**Table S3 Volumes of all Targets and organs at risk on each scan**

|  | **ATS Plan (n=85)**  **Median (range)** | | **PV scan (n=85)**  **Median (range)** | | **Beam-on scan (n=49)**  **Median (range)** | | **Post scan (n=71)**  **Median (range)** | |
| --- | --- | --- | --- | --- | --- | --- | --- | --- |
| **PTV volume (cc)** | 91.45 | (57.25—137.52) | 91.67 | (56.29—138.98) | 90.74 | (57.10—138.91) | 90.71 | (58.45—134.86) |
| **Δ PTV volume (cc)** |  |  | 0.22 | (-4.0—6.22) | 0.18 | (-3.12—3.47) | -0.44 | (-3.06—-2.70) |
| **CTV volume (cc)** | 58.29 | (34.89—94.95) | 58.60 | (34.21—95.94) | 58.67 | (34.73—94.66) | 58.11 | (35.26—94.61) |
| **Δ CTV volume (cc)** |  |  | 0.31 | (-2.96—2.94) | 0.25 | (-1.70—2.71) | 0.10 | (-2.07—2.61) |
| **Prostate volume (cc)** | 39.25 | (22.98—66.71) | 39.39 | (22.41—67.99) | 40.75 | (22.78—67.56) | 38.72 | (22.85—65.75) |
| **Δ Prostate (cc)** |  |  | 0.14 | (-1.22—2.04) | 0.10 | (-1.49—2.33) | 0.10 | (-1.85—1.92) |
| **CTV4000 volume (cc)** | 30.81 | (17.47—55.36) | 30.91 | (16.97—56.46) | 31.71 | (17.28—55.87) | 30.55 | (17.32—56.10) |
| **Δ CTV4000 volume (cc)** |  |  | 0.10 | (-3.06—3.03) | 0.09 | (-1.75—1.79) | 0.30 | (-3.32—4.94) |
| **Rectum volume (cc)** | 39.94 | (19.15—84.33) | 41.65 | (21.38—85.47) | 43.23 | (27.73—84.18) | 42.07 | (25.05—79.31) |
| **Δ Rectum volume (cc)** |  |  | 1.59 | (-7.39—34.41) | 2.37 | (-22.58—40.10) | 1.37 | (-25.03—34.20) |
| **Bladder volume (cc)** | 172.74 | (58.55—420.45) | 256.55 | (74.89—592.38) | 312.34 | (81.50—630.13) | 310.27 | (91.93—650.67) |
| **Δ Bladder volume (cc)** |  |  | 83.96 | (4.11—343.02) | 136.75 | (19.18—363.88) | 140.36 | (-0.91—405.83) |

**Δ: Volume Differences compared to The Corresponding Original ATS Plan**
