# Supplementary figures and images for: Assessment of delivered dose in prostate cancer patients treated with ultra-hypofractionated radiotherapy on 1.5-Tesla MR-Linac
Source: Front Oncol. 2023 Jan 19;13:1039901. doi: 10.3389/fonc.2023.1039901 (PMC9893501; doi:10.3389/fonc.2023.1039901)

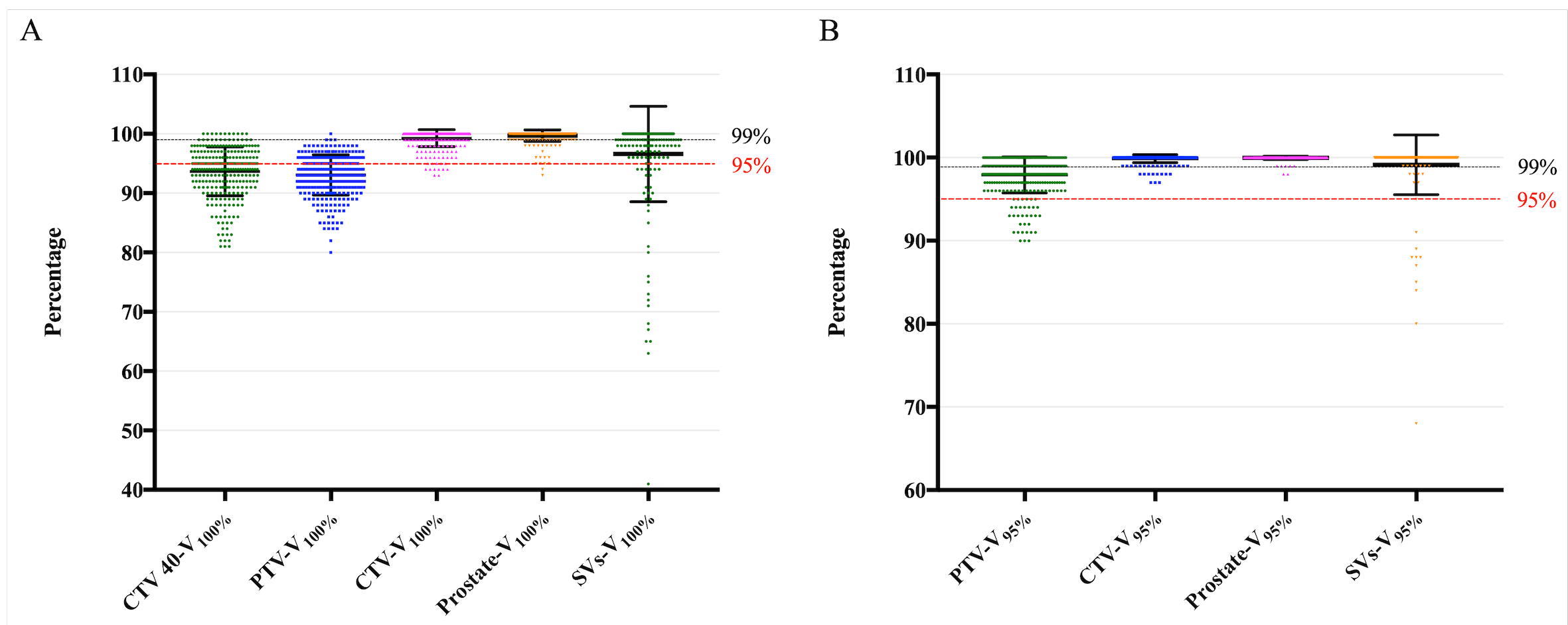

Supplement: Supplementary Figure 1 — Overviews of planning targets of all fractions, calculated by the daily ATS plan dose in PV-, Bn- and post-MR scans. 100% prescription dose (A) and 95% prescription dose (B). Individual data points are shown as dots. The mean ± SD are shown as the error bars. [file Image_1.tiff]

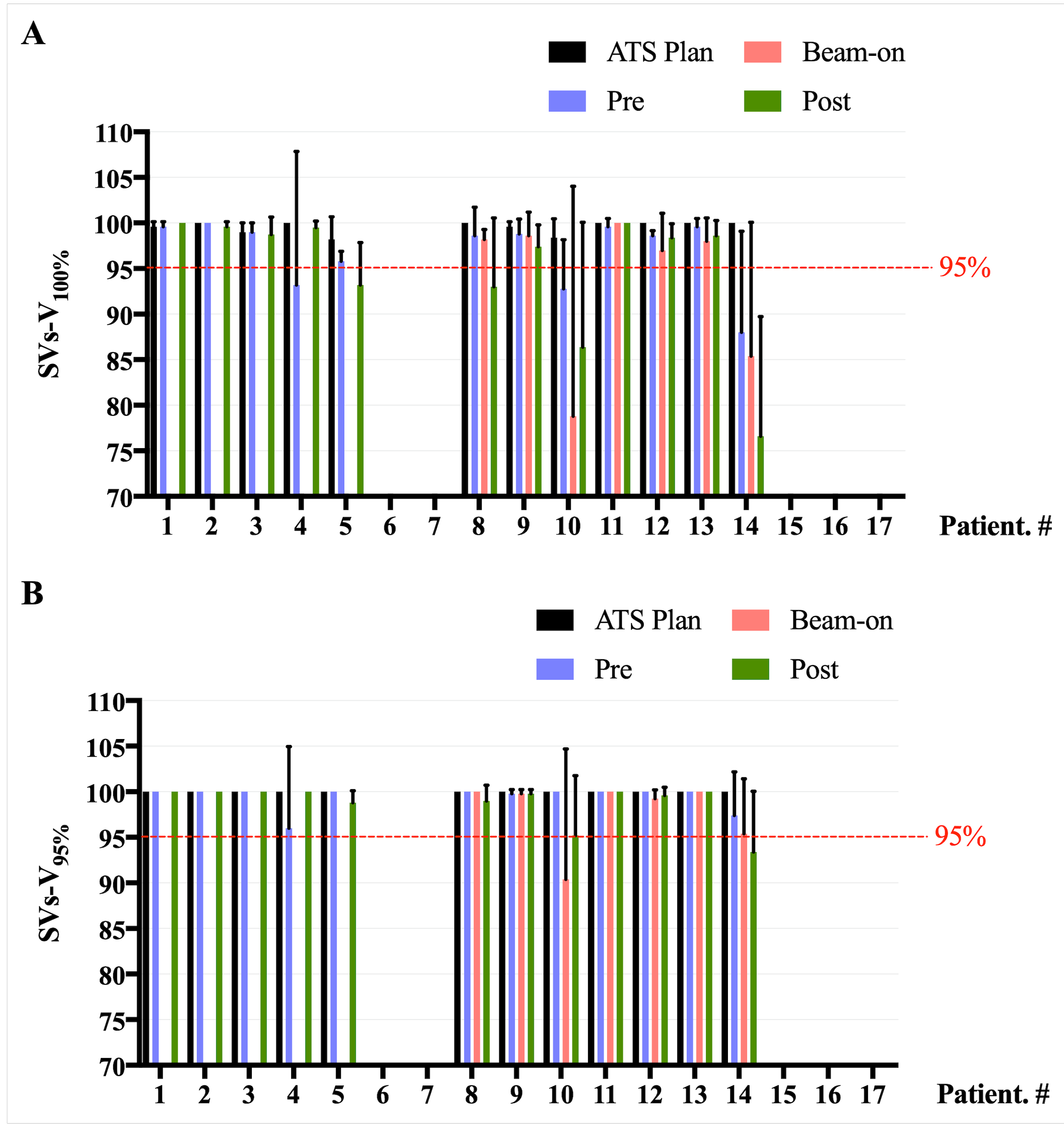

Supplement: Supplementary Figure 2 — Per-patient SVs-V100% (A) and SVs-V95% (B) mean values to the clinical target volume (CTV) summed by five fractions of pre- (ATS plan), PV-, Bn- and post-MR scans. Scar bars present the standard deviation (SD). No beam-on scans were acquired for the first 7 patients due to concerns about unexpected target and OAR moving. No SVs irradiation for Patient. #6, #7, #15 to #17. [file Image_2.tiff]
